# Analyses of lncRNA and mRNA profiles in recurrent atrial fibrillation after catheter ablation

**DOI:** 10.1186/s40001-024-01799-3

**Published:** 2024-04-20

**Authors:** Huaiguang Tang, Kongmiao Lu, Yan Wang, Yue Shi, Wansheng Ma, Xiaomeng Chen, Bingong Li, Yibing Shao

**Affiliations:** 1 Department of Cardiology, Qingdao Municipal Hospital, Qingdao University, No. 5, Donghai Middle Road, Qingdao, 266071 Shandong China; 2 Department of Pulmonary and Critical Care Medicine, Qingdao Municipal Hospital, University of Health and Rehabilitation Sciences, No. 5, Donghai Middle Road, Qingdao, 266071 Shandong China; 3 Department of Cardiology, Qingdao Municipal Hospital, University of Health and Rehabilitation Sciences, No. 5, Donghai Middle Road, Qingdao, 266071 Shandong China

**Keywords:** Recurrent atrial fibrillation, lncRNA, mRNA, RNA sequencing, lncRNA‒mRNA regulatory network

## Abstract

**Background:**

Atrial fibrillation (AF) is the most common cardiac arrhythmia worldwide. Catheter ablation has become a crucial treatment for AF. However, there is a possibility of atrial fibrillation recurrence after catheter ablation. Our study sought to elucidate the role of lncRNA‒mRNA regulatory networks in late AF recurrence after catheter ablation.

**Methods:**

We conducted RNA sequencing to profile the transcriptomes of 5 samples from the presence of recurrence after AF ablation (P-RAF) and 5 samples from the absence of recurrence after AF ablation (A-RAF). Differentially expressed genes (DEGs) and long noncoding RNAs (DE-lncRNAs) were analyzed using the DESeq2 R package. The functional correlations of the DEGs were assessed through Gene Ontology (GO) and Kyoto Encyclopedia of Genes and Genomes (KEGG) analyses. A protein‒protein interaction (PPI) network was constructed using STRING and Cytoscape. We also established a lncRNA‒mRNA regulatory network between DE-lncRNAs and DEGs using BEDTools v2.1.2 software and the Pearson correlation coefficient method. To validate the high-throughput sequencing results of the hub genes, we conducted quantitative real-time polymerase chain reaction (qRT‒PCR) experiments.

**Results:**

A total of 28,528 mRNAs and 42,333 lncRNAs were detected. A total of 96 DEGs and 203 DE-lncRNAs were identified between the two groups. GO analysis revealed that the DEGs were enriched in the biological processes (BPs) of “regulation of immune response” and “regulation of immune system process”, the cellular components (CCs) of “extracellular matrix” and “cell‒cell junction”, and the molecular functions (MFs) of “signaling adaptor activity” and “protein–macromolecule adaptor activity”. According to the KEGG analysis, the DEGs were associated with the “PI3K–Akt signaling pathway” and “MAPK signaling pathway.” Nine hub genes (*MMP9*,* IGF2*, *FGFR1*, *HSPG2*, *GZMB*, *PEG10*, *GNLY*, *COL6A1*, and *KCNE3*) were identified through the PPI network. lncRNA-TMEM51-AS1-201 was identified as a core regulator in the lncRNA‒mRNA regulatory network, suggesting its potential impact on the recurrence of AF after catheter ablation through the regulation of COL6A1, FGFR1, HSPG2, and IGF2.

**Conclusions:**

The recurrence of atrial fibrillation after catheter ablation may be associated with immune responses and fibrosis, with the extracellular matrix playing a crucial role. TMEM51-AS1-201 has been identified as a potential key target for AF recurrence after catheter ablation.

**Supplementary Information:**

The online version contains supplementary material available at 10.1186/s40001-024-01799-3.

## Introduction

Atrial fibrillation (AF) is the most common cardiac arrhythmia in the world [1]. It has numerous complications, including stroke, heart failure, cognitive impairment, and cardiac arrest [2]. These complications lead to decreased patient quality of life, increased mortality rates, and increased health care costs [1]. The primary objectives in treating patients with AF include symptom amelioration, heart rate or rhythm control, and stroke risk reduction [3]. Catheter ablation is now the preferred curative treatment for atrial fibrillation (AF) [4]. However, catheter ablation surgery for AF has the potential for recurrence [5]. The success rate of ablation in patients with paroxysmal AF is approximately between 60 and 79% [6]. However, for long-standing persistent AF patients, the success rate of isolated pulmonary vein isolation treatment alone ranges from 36 to 56% [7, 8]. The recurrence of AF after catheter ablation can be partially attributed to pulmonary vein reconnection [9], but the specific underlying mechanisms remain unclear.

LncRNAs are a group of nonprotein-coding transcripts with lengths exceeding 200 nucleotides [10], and they play crucial roles in various diseases [11, 12]. Research has shown that lncRNAs play a crucial role in AF. They participate in AF by regulating processes such as atrial fibrosis and inflammatory responses [13]. Additionally, specific lncRNAs, such as MALAT1, can serve as biomarkers to predict the recurrence of AF after catheter ablation [14, 15].

Currently, clinical research predominantly focuses on the risk factors and prognostic markers associated with recurrence following catheter ablation for AF. However, the mechanisms underlying the recurrence of AF after catheter ablation remain unclear. To the best of our knowledge, there has been no prior investigation into the mechanisms of AF recurrence after catheter ablation through the analysis of lncRNA‒mRNA regulatory networks. The primary objectives of this study were to investigate the mechanisms underlying the recurrence of AF after catheter ablation and to analyze the lncRNA‒mRNA regulatory networks involved. Our research aimed to fill the gap in understanding the recurrence of AF postablation.

Through a series of bioinformatics analyses and qRT‒PCR validation, we identified correlations between the recurrence of AF after catheter ablation and pathways related to immune inflammation and fibrotic processes. By constructing a hub gene network, we identified vital nodes that play a critical role in the recurrence process. Furthermore, we established a lncRNA/mRNA network associated with postcatheter ablation AF recurrence. In summary, we conducted a comprehensive RNA-level analysis of postcatheter ablation AF recurrence, including the identification of crucial pathways, selection of hub genes, construction of RNA regulatory networks, and validation through qRT‒PCR.

## Methods

### Study population and specimen collection

Long-term follow-up was conducted on patients who underwent catheter ablation surgery at Qingdao Municipal Hospital from 2019 to 2021. Patients with hyperthyroidism, autoimmune diseases, valvular heart diseases, infective endocarditis, severe liver or kidney dysfunction, or malignancies were excluded. From the eligible patients, we selected 5 individuals who had an absence of recurrence after AF ablation (A-RAF) and 5 who had the presence of recurrence after AF ablation (P-RAF) for inclusion in the study. Detailed patient information is presented in Table 1.

**Table 1:** Baseline characteristics of study participants

	SR (*n* = 5)	AF(*n* = 5)	P value
Age (years)	70.80 ± 3.768	70.20 ± 4.658	0.796
Height (m)	1.668 ± 0.10183	1.646 ± 0.11929	0.535
Weight (kg)	76.1 ± 6.30872	72 ± 17.63519	0.151
BMI (kg/m^2^)	27.5823 ± 4.04698	26.377 ± 4.02888	0.905
Female (%)	60	40	
HTN (%)	100	80	
CAD (%)	20	20	
CHA2DS2_VASc	4.4 ± 1.67332	3.8 ± 2.16795	0.418
LAD (mm)	42.60 ± 4.037	41.20 ± 5.718	0.125
RAD (mm)	36.80 ± 3.194	37.00 ± 5.244	0.387
LVEF (%)	57.40 ± 2.702	57.20 ± 3.114	0.505
WBC (10^9^/L)	6.512 ± 1.76967	5.99 ± 1.76724	0.827
Neutrophil (10^9^/L)	4.246 ± 1.32668	3.06 ± 1.09501	0.426
Lymphocyte (10^9^/L)	1.704 ± 0.7451	2.188 ± 0.45593	0.095
Monocyte (10^9^/L)	0.47 ± 0.18561	0.528 ± 0.21017	0.421
Hemoglobin (10^9^/L)	133.2 ± 13.10343	135.6 ± 19.85699	0.354
Platelet (10^9^/L)	208.6 ± 30.54996	206.8 ± 31.16408	0.887

*BMI* Body Mass Index, *HTN* Hypertension, *CAD* coronary artery disease, *LAD* left atrium diameter, *RAD* right atrium diameter, *LVEF* left ventricular ejection fraction, *WBC* white blood cell

Venous blood was collected from each participant using PAXgene Blood RNA Tubes (Qiagen, CA, USA) to preserve the gene expression profile. All patients provided informed consent, and their records and information were anonymized before analysis. This study was conducted following the guidelines approved by the Clinical Research Ethics Committee of Qingdao Municipal Hospital.

### RNA extraction and cDNA synthesis

The total RNA of each sample was extracted from whole venous blood with the PAXgene Blood RNA Kit (PreAnalytiX, Qiagen/BD) and fractionated by size. RNA purity and concentration were assessed using a NanoDrop 2000 spectrophotometer to ensure the quality of the RNA samples. Additionally, RNA integrity was evaluated using an Agilent 2100/LabChip GX to confirm that the RNA was suitable for downstream analyses. After RNA preparation, the Illumina^®^ libraries were quantified using a Qubit 3.0 fluorescence quantifier and then further quantified accurately by qRT‒PCR. Subsequently, PE150 mode sequencing of the Illumina^®^ library was performed on the Illumina NovaSeq 6000 sequencing platform. Sequence reads from large RNAs were aligned with the human genome (*Homo sapiens*. GRCh38_release95.genome.fa) using HISAT software and assembled with StringTie software.

### Screening for differentially expressed genes (DEGs) and differentially expressed long noncoding RNAs (DE-lncRNAs)

Before conducting the differential gene expression analysis, read counts for each sequenced library were adjusted using the edgeR program package with a single scaling normalization factor. Differential expression analysis was performed on the ten samples using the EBseq (2010) R package. A statistical significance threshold of |fold change|≥ 1.5 and *p* < 0.01 was applied.

### DEG functional enrichment analysis

To investigate the functions of the DEGs, we conducted Gene Ontology (GO) and Kyoto Encyclopedia of Genes and Genomes (KEGG) pathway enrichment analyses. These analyses were performed using the Database for Annotation Visualization and Integrated Discovery (DAVID) online tool. The cutoff values were set as a *p* value < 0.05 and a gene count per annotated pathway ≥ 2 [16]. The results were visualized using the ggplot2 R package.

### Protein‒protein interaction (PPI)network construction and hub gene identification

We used the STRING database to construct the PPI network of the intersecting DEGs [17]. These intersecting DEGs were imported into Cytoscape v3.9.1, and the hub genes were identified using the Degree algorithm in CytoHubba. To ensure the quality of the hub genes, the selection criteria were set at |fold change|≥ 2 and *p* < 0.01.

### LncRNA cis- and trans-target gene prediction analysis

To identify the DEGs targeted by the DE-lncRNAs, we conducted both cis- and trans-target analyses. For cis-regulatory interactions, we used the window function within BEDTools v2.1.2 software to identify cis-target genes located within a range of 100 kb upstream and downstream of the differentially expressed lncRNAs. The potential for lncRNAs to act in a cis-regulatory manner was subsequently assessed. For trans-regulatory interactions, we employed the Pearson correlation coefficient method to analyze the correlation between lncRNAs and mRNAs across samples, selecting genes with r values ≥ 0.9 and *p* < 0.05 as the trans-target genes of lncRNAs.

### Quantitative real-time polymerase chain reaction confirmation

We used the TUREscript 1st Strand cDNA Synthesis Kit (Aidlab) for reverse transcription of the total RNA. The sequences of the primers used can be found in Additional file 1: Table S1. Detection was conducted using an Analytik Jena-qTOWER 2.2 fluorescence quantitative PCR instrument (Germany). The qRT‒PCR results were analyzed using Student's *t* test, with *p* < 0.05 indicating statistical significance.

### Analysis of immune cells

The CIBERSORT R package was used to analyze the distribution of immune cells in the RNA-seq data and to compare the differences in immune cell composition between the P-RAF group and the A-RAF group. The results were visualized using the ggplot2 R package, and the accuracy of the immune cell analysis was confirmed by comparing the results with blood cell count data.

### Statistical analysis

Statistical analysis was performed using SPSS software (version 23; SPSS). The data are presented as the means ± SDs. We assessed the normality of the data using SPSS. Subsequently, for normally distributed data, we employed Student’s *t* test to assess the significance of differences in means. For data that did not follow a normal distribution, we utilized the Wilcoxon rank-sum test to determine the significance of differences in medians. A *p* value < 0.05 was considered to indicate a significant difference.

## Results

### Transcriptome results

In our Illumina NovaSeq 6000 transcriptome sequencing data, 28,528 mRNAs and 42,333 lncRNAs were detected.

### Identification of DEGs and DE-lncRNAs

**Fig. 1:**
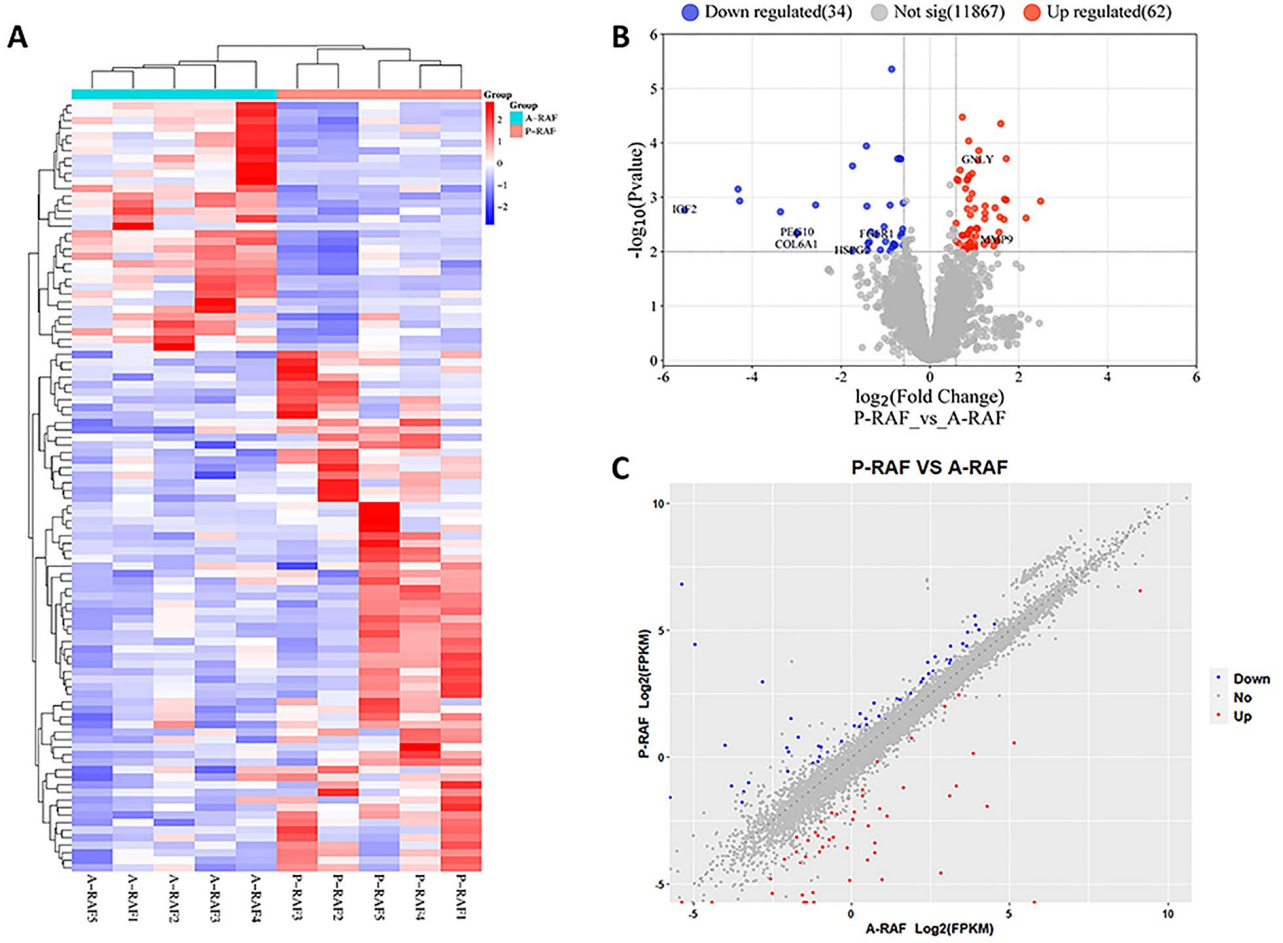
Differentially expressed mRNAs in the P-RAF group and A-RAF group. **A** Hierarchical clustering showing a difference in the mRNA expression profile between the two groups and homogeneity within groups. **B** Volcano plot of the differentially expressed mRNAs. The red points (high level) and blue points (low level) indicate the differentially expressed mRNAs with statistical significance. The threshold was set as FDR < 0.01 and |log2 (fold change)|≥ 1.5. **C** Scatter plot of mRNA expression. The mRNAs are indicated as red points (high level) and blue points (low level). The mRNA fold changes (FCs) between the P-RAF group and the A-RAF group exceeded 1.5

**Fig. 2:**
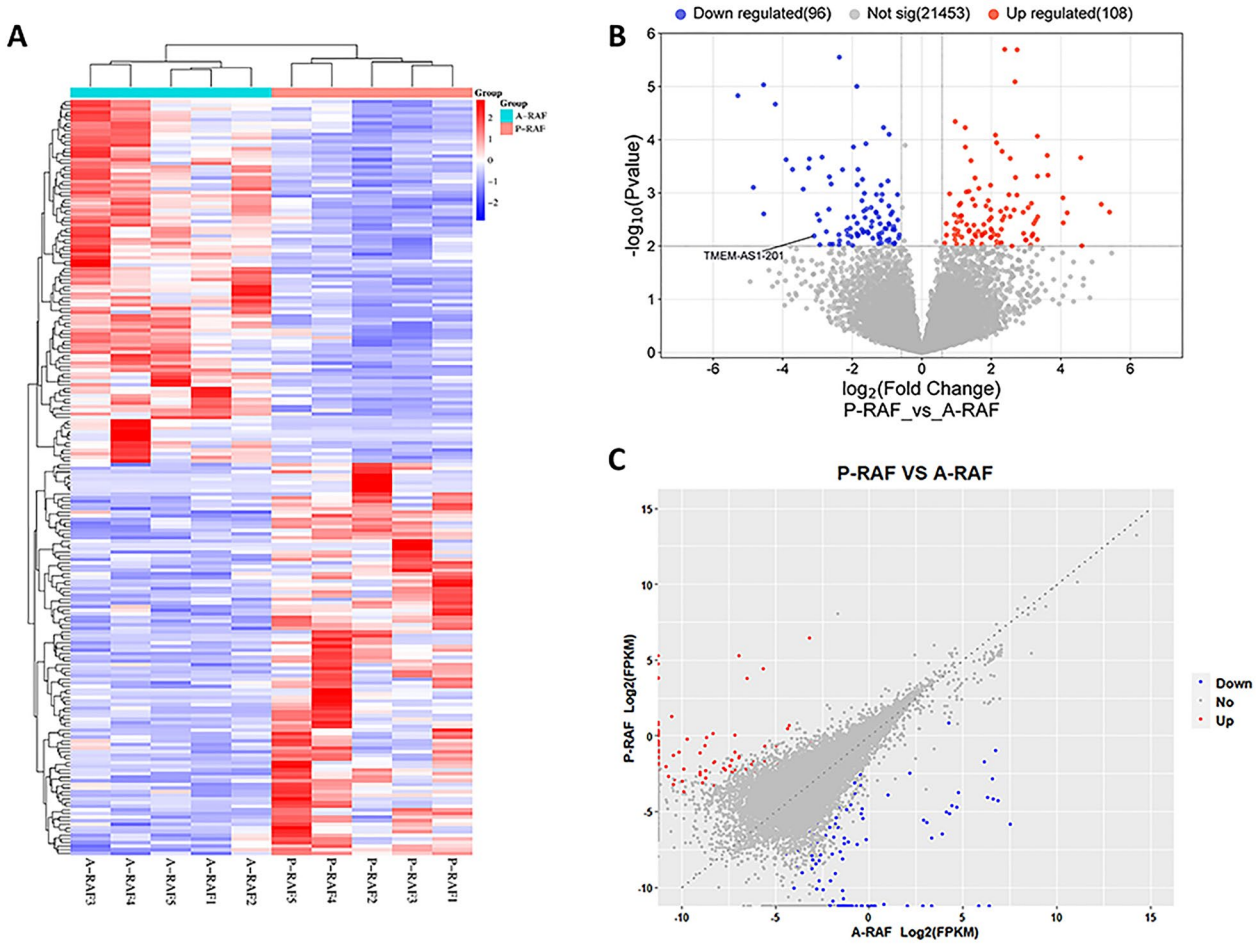
Differentially expressed lncRNAs in the P-RAF group and A-RAF group. **A** Hierarchical clustering showing a difference in the lncRNA expression profile between the two groups and homogeneity within groups. **B** Volcano plot of the differentially expressed lncRNAs. The red points (high level) and blue points (low level) indicate the differentially expressed mRNAs with statistical significance. The threshold was set as FDR < 0.01 and |log2 (fold change)|≥ 1.5. **C** Scatter plot of lncRNA expression. The lncRNAs are indicated as red points (high level) and blue points (low level). The lncRNA fold changes (FCs) between the P-RAF group and the A-RAF group exceeded 1.5

We utilized the EdgeR software package to identify 96 differentially expressed genes (DEGs) between P-RAF and A-RAF. Among these genes, 62 were upregulated, and 34 were downregulated. Using the same methodology, we also identified 203 differentially expressed lncRNAs (DE-lncRNAs), 107 of which were upregulated and 96 of which were downregulated. The volcano plot and heatmap for DEGs are displayed in Fig. 1, and the volcano plot and heatmap for DE-lncRNAs are presented in Fig. 2. The lists of DEGs and DE-lncRNAs can be found in Additional file 1: Tables S2 and S3, respectively.

### Biological functions of DEGs

**Fig. 3:**
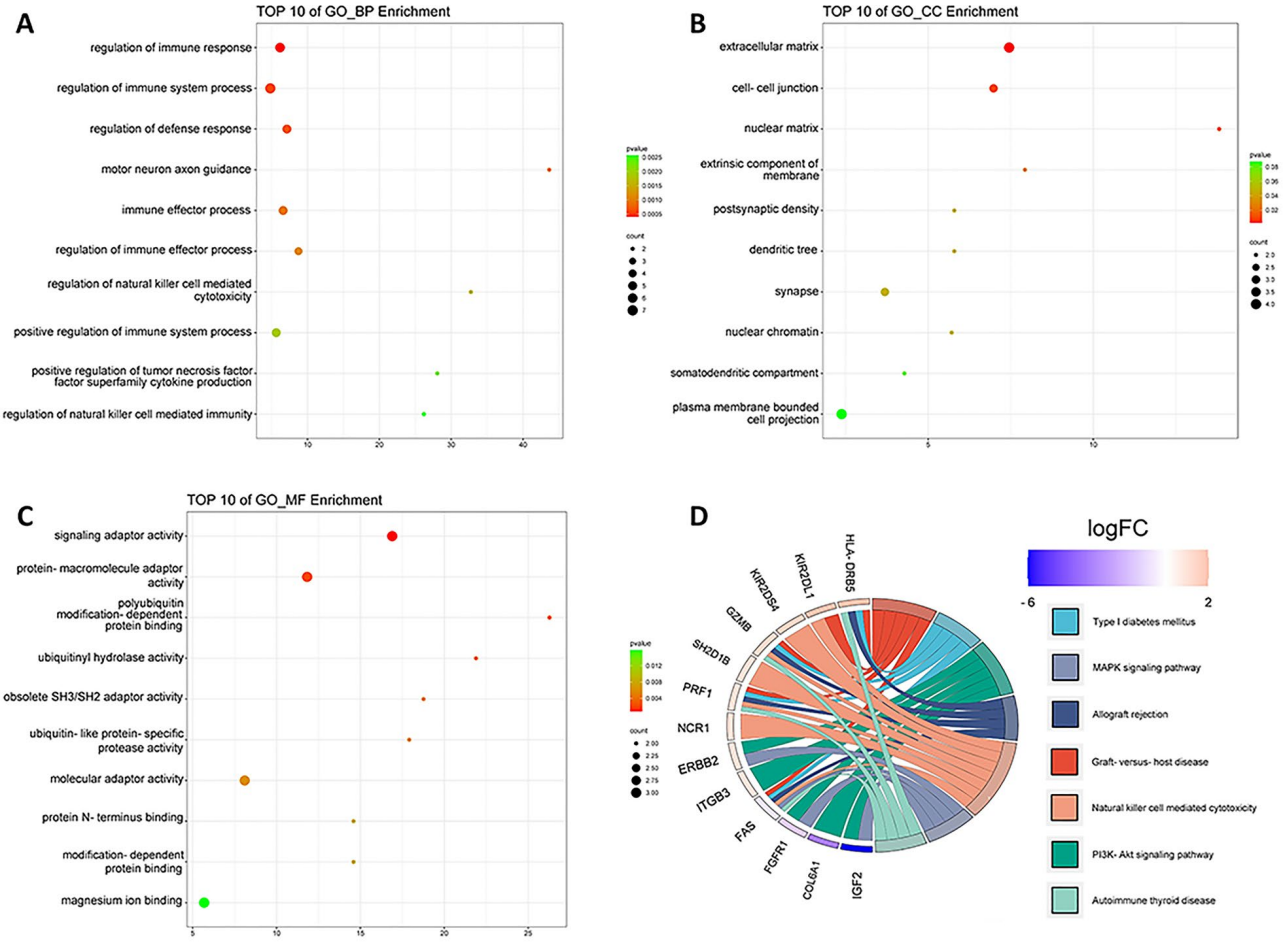
Functional enrichment analysis of DEGs. **A–C** Partial visualization of GO analysis of the biological process, cellular component, and molecular function categories for node genes. The *X*-axis represents the enrichment factor, and the *Y*-axis represents different ontologies. The colors and sizes of the dots indicate *p* values and the number of genes, respectively. **B** KEGG pathway analysis of node genes. The left side displays genes, while the right side depicts significantly enriched pathways. "Type I diabetes mellitus" is represented in sky blue, "MAPK signaling pathway" in deep gray, "Allograft rejection" in navy blue, "Graft-versus-host disease" in red, "Natural killer cell mediated cytotoxicity" in orange, "PI3K-Akt pathway" in dark green, and "Autoimmune thyroid disease" in light green. These colors correspond to the respective pathways. The connections between genes and pathways indicate that genes were enriched in related pathways. GO, Gene Ontology; KEGG, Kyoto Encyclopedia of Genes and Genomes

Functional enrichment analysis was conducted for 96 differentially expressed genes. The DAVID database was used for conducting KEGG analysis and GO analysis. GO analysis revealed that DEGs were primarily enriched in the biological processes (BPs) of “regulation of immune response,” “regulation of immune system process,” “regulation of defense response,” “motor neuron axon guidance,” and “immune effector process” (Fig. 3A), the cellular components (CCs) of “extracellular matrix,” “cell‒cell junction,” and “nuclear matrix” (Fig. 3B), and the molecular functions (MFs) of “signaling adaptor activity,” “protein–macromolecule adaptor activity,” “polyubiquitin modification-dependent protein binding,” and “ubiquitinyl hydrolase activity” (Fig. 3C). The DEGs were significantly enriched in the PI3K–Akt signaling pathway and MAPK signaling pathway according to the KEGG results (Fig. 3D).

### Protein‒protein interaction analysis

**Fig. 4:**
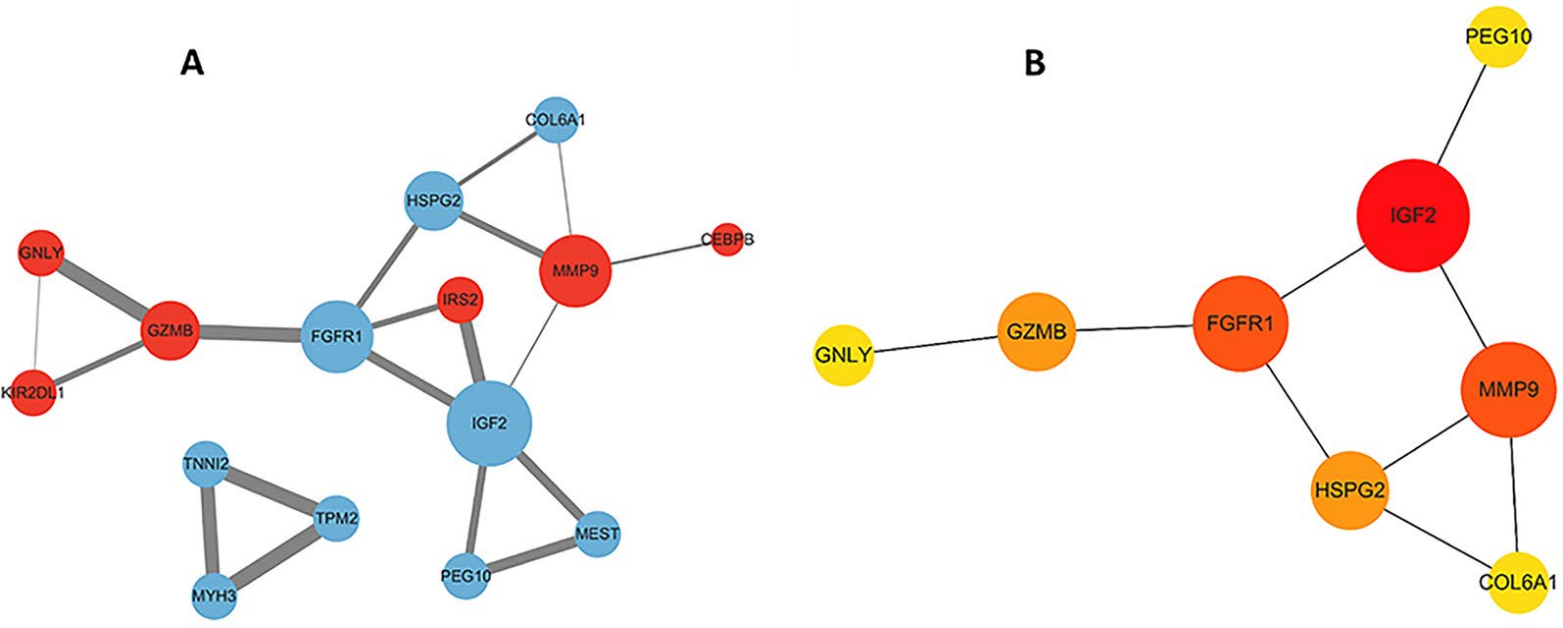
Interaction network and hub gene network. **A** Protein‒protein interaction network. Among the 57 DEGs, 15 were confirmed to have mutual regulatory relationships. Red indicates upregulation, and blue indicates downregulation. The size of the nodes is related to the number of connections; the larger the number of connections is, the larger the size of the node. **B** Hub gene network. The PPI network graph was simplified using the degree algorithm to identify hub genes. Eight genes (*IGF2*, *FGFR1*, *MMP9*, *HSPG2*, *GZMB*, *PEG10*, *GNLY*, and *COL6A1*) were identified as the top candidates. The colors of the nodes in the graph indicate different levels of importance, ranging from yellow to red

The PPI network of DEGs was constructed using data retrieved from the STRING database. Fifteen protein-coding genes were identified in the PPI network and visualized via Cytoscape (Fig. 4A). The nodes in the network were analyzed using the degree algorithm from the cytoHubba plugin, and a total of 8 genes were identified as hub genes, namely, *IGF2*, *FGFR1*, *MMP9*, *HSPG2*, *GZMB*, *PEG10*, *GNLY* and *COL6A1* (Fig. 4B).

### LncRNA–mRNA regulatory network

**Fig. 5:**
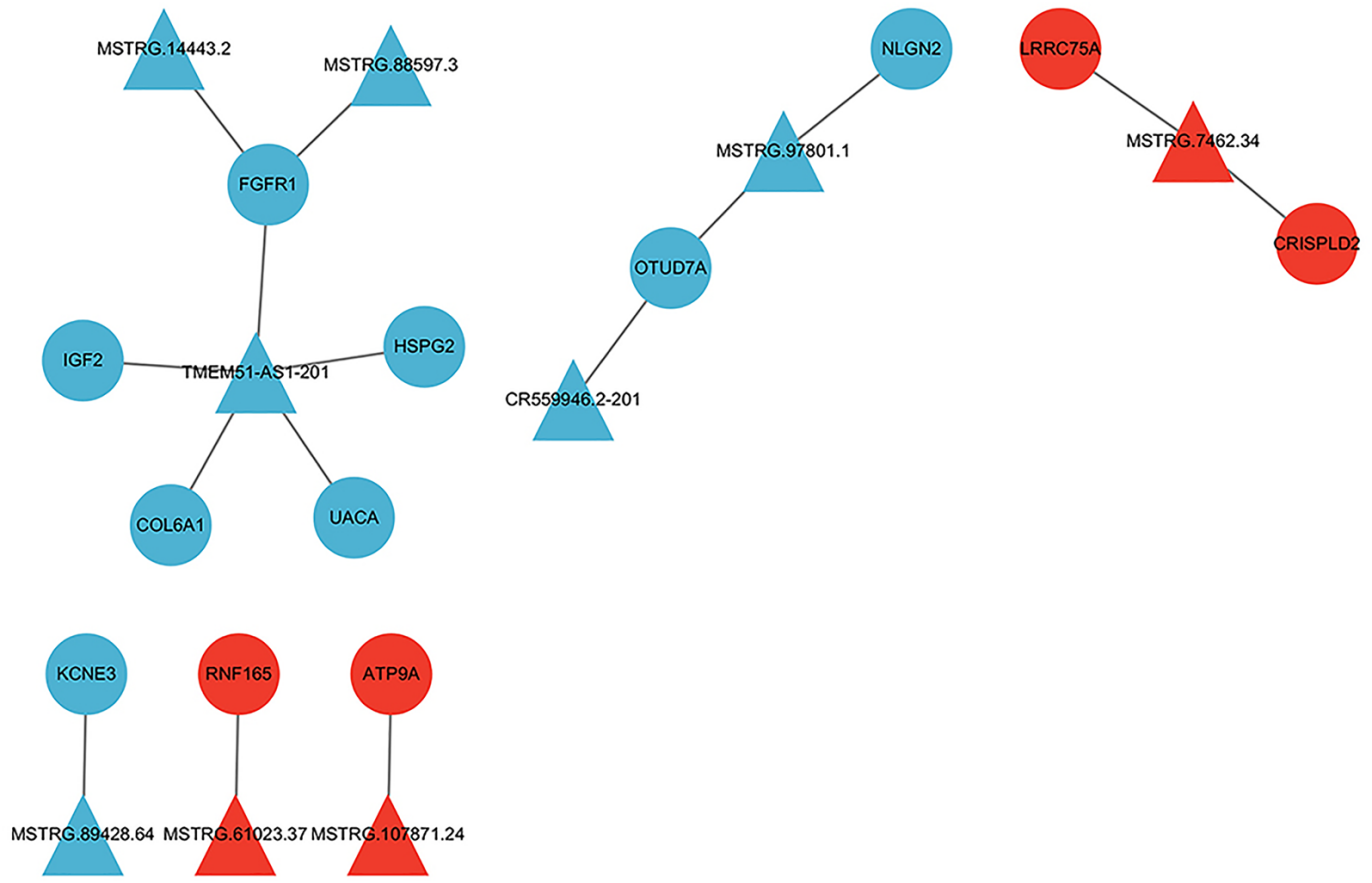
DElncRNA–DEmRNA relationship graph. Triangles indicate lncRNAs, circles indicate mRNAs, blue indicates downregulated genes, and red indicates upregulated genes

By predicting DElncRNA–DEmRNA target relationships using various modes, including cis-regulation and complementary base pairing principles, we identified 15 pairs of regulated DElncRNA–DEmRNA relationships. Subsequently, we visualized these relationships using Cytoscape software (Fig. 5). In the graph, TMEM-AS1-201 emerged as a key node that regulates 5 differentially expressed genes: *FGFR1*, *IGF2*, *COL6A1*, *UACA*, and *HSPG2*. Upon comparing these 5 genes with the list of hub genes, it was evident that *FGFR1*, *IGF2*, *COL6A1*, and *HSPG2* are hub genes. These findings imply that TMEM-AS1-201 may serve as a critical locus in the recurrence of AF after catheter ablation.

### Validation of differentially expressed lncRNAs and mRNAs

**Fig. 6:**
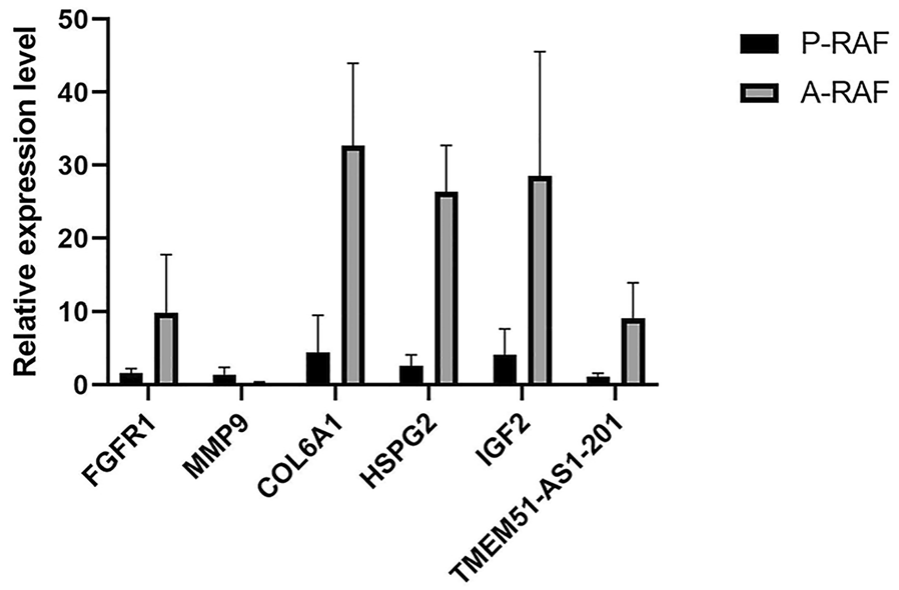
qRT‒PCR validation of differentially expressed lncRNAs and mRNAs

We conducted qRT‒PCR validation of 8 hub genes and one lncRNA (TMEM-AS1-201) (Fig. 6). In the P-RAF group, *MMP9*, *GZMB*, and *GNLY* were upregulated compared to those in the A-RAF group, and *IGF2*, *FGFR1*, *HSPG2*, *PEG10*, *COL6A1*, and TMEM51-AS1-201 were downregulated compared to those in the A-RAF group. These results demonstrate good consistency between the high-throughput sequencing and qRT‒PCR results.

### Analysis of immune cells

**Fig. 7:**
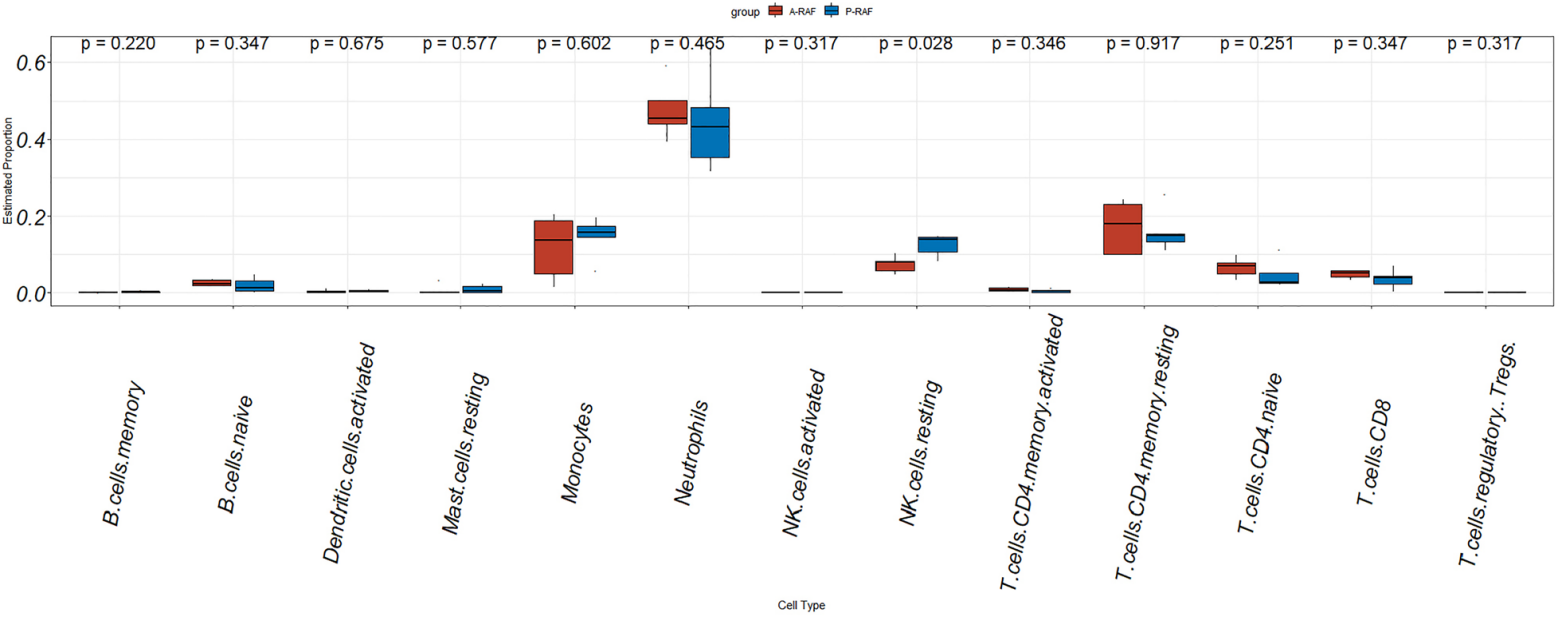
Immune analysis box plot. The *X*-axis represents the types of immune cells, and the *Y*-axis represents the proportion of these immune cells among all immune cells. The red bars indicate the A-RAF group, and the blue bars indicate the P-RAF group. “NK cell resting” is elevated was the P-RAF group, and the difference was statistically significant

Using CIBERSORT analysis, we predicted the proportions of immune cells and observed a significantly higher proportion of "NK cell resting" cells in the P-RAF group compared to the A-RAF group (Fig. 7). These predictions were consistent with the blood cell count results obtained from the hospital, indicating the reliability of the CIBERSORT predictions.

## Discussion

AF is the most common type of arrhythmia worldwide [18]. Catheter ablation is the primary method for treating AF, but it has a high recurrence rate. Research on AF recurrence after catheter ablation has primarily concentrated on factors such as ablation methods, atrial size, and postablation edema, which can explain early recurrences [19]. However, the mechanisms underlying the late recurrence of AF after catheter ablation remain unclear. In this study, we discovered that the recurrence of AF after catheter ablation may be associated with immune responses and myocardial fibrosis resulting from extracellular matrix remodeling.

The role of factors such as myocardial fibrosis caused by extracellular matrix accumulation and immune reactions in AF development has been widely recognized [20, 21]. An increasing number of studies indicate a close association between immune inflammatory responses and AF. Previous research has demonstrated increased macrophage accumulation in the atria of patients with AF [22]. Canpolat et al. [23] reported that the ratio of neutrophils to lymphocytes before and after ablation can predict the recurrence rate after ablation. Additionally, there is a close association between the recurrence of AF after catheter ablation and the extracellular matrix [24]. Berg et al. [25] reported that extracellular matrix remodeling occurs before the onset of AF, suggesting that extracellular matrix remodeling is a direct cause of AF occurrence.

In our research, we performed high-throughput transcriptome analysis on venous blood samples collected from two distinct patient groups: the P-RAF group and the A-RAF group. Through GO-BP pathway enrichment analysis, we revealed that postcatheter ablation AF recurrence is associated with “immune regulation pathways” and the “regulation of immune system process”. Subsequently, the analysis of immune cells revealed a significant increase in the “NK cell resting” population in the P-RAF group. Natural killer (NK) cells possess a variety of functions. On the one hand, they exhibit natural cytotoxicity and can kill other cells [26]. On the other hand, they have a regulatory role in modulating other immune cells and promoting tissue growth [27, 28]. These unique functions play crucial but often contradictory roles in different diseases [28]. In the context of myocardial infarction, it has been established that NK cells regulate neutrophil involvement in chemokine clearance through the secretion of IFN-γ. This process halts neutrophil migration to the infarcted myocardium, thereby helping to suppress ongoing inflammatory responses [27, 29]. These findings suggest that NK cells may influence the recurrence of AF after catheter ablation by modulating immune regulatory pathways.

PPI analysis was subsequently performed on the differentially expressed genes. Nine differentially expressed genes, namely, *IGF2*, *FGFR1*, *MMP9*, *HSPG2*, *GZMB*, *PEG10*, *GNLY*, *COL6A1*, and *KCNE3*, were identified as hub genes. Among the hub genes, MMP-9 had the closest association with AF. MMP-9 is a member of the matrix metalloproteinase family. Research has shown that MMP-9 can participate in the development of various heart diseases. During the process of AF, MMP-9 promotes the occurrence of atrial remodeling [30]. As AF progresses, the serum levels of MMP-9 gradually increase. Therefore, MMP-9 can serve as a biomarker for AF [31]. In obese patients, MMP-9 can be used to predict the occurrence of paroxysmal AF [32]. We detected differences in the expression of *MMP-9* between the two groups (P-RAF and A-RAF). As a key node in protein‒protein interaction (PPI) networks, MMP-9 may play an important role in AF recurrence after catheter ablation, but further exploration is needed.

We performed a combined analysis of the hub genes and the ceRNA network to obtain the ceRNA regulatory network associated with the hub genes. This network comprises four hub genes, namely, *COL6A1*, *FGFR1*, *HSPG2*, and *IGF2*, and one lncRNA, TMEM51-AS1-201. Fibroblast growth factor receptor 1 (FGFR1) is a transmembrane receptor protein belonging to the family of receptor tyrosine kinases. FGFR1 can further regulate the PPARγ pathway through FGF21 [33]. Previous research has confirmed that PPARγ possesses anti-myocardial fibrosis functionality [34].

Insulin-like growth factor 2 (IGF-2) is a synthetic growth factor that promotes cell proliferation and migration. Previous research has indicated that IGF-2 is widely distributed in muscle tissue and is involved in various biological processes [35]. IGF-2 participates in creating a fibrotic environment both intracellularly and extracellularly, promoting the progression of fibrotic diseases [36]. Notably, IGF-2 has also been associated with anti-inflammatory effects. It can polarize macrophages toward an anti-inflammatory phenotype and acquire anti-inflammatory characteristics [37]. In a separate study, injecting recombinant human IGF-2 into mice suppressed inflammation and increased the number of anti-inflammatory cells [38]. Previous research has established a significant link between AF recurrence after catheter ablation and atrial fibrosis and the inflammatory response. Our study revealed higher RNA levels of *IGF-2* in patients who maintained sinus rhythm after ablation than in those who experienced AF recurrence postablation. These findings suggest that the anti-inflammatory effect of IGF-2 might have a more significant impact than its fibrosis-promoting effect, potentially inhibiting the occurrence of AF. Currently, there is a lack of research on the impact of IGF-2 on AF occurrence, and considering its widespread distribution and regulatory functions in muscle tissue, further exploration of its role in the process of AF recurrence after catheter ablation is warranted.

Through the analysis of the lncRNA‒mRNA regulatory network, we identified genes within the network that are associated with immune responses and fibrosis. In the GO–CC pathway enrichment analysis, we observed significant enrichment of “extracellular matrix”, which is a critical process in fibrosis. Thus, it can be inferred that the recurrence of AF after catheter ablation may be closely associated with immune responses and myocardial fibrosis, with the extracellular matrix possibly playing a pivotal role in the AF recurrence process.

In the lncRNA‒mRNA regulatory network, “TMEM51-AS1-201” was indicated as the core and can regulate 4 hub genes, *FGFR1* and *IGF2*, which have been previously confirmed to be closely associated with immune responses and fibrosis. These findings suggest that “TMEM51-AS1-201” may play a crucial role in AF recurrence after catheter ablation by modulating immune responses and fibrosis. Thus, it could be considered a key target for AF recurrence after catheter ablation.

Our study has several limitations worth discussing. First, conducting in vivo and in vitro experiments to validate the biological functions of the hub genes could provide valuable insights. Second, increasing the sample size may yield more comprehensive information. Third, “TMEM51-AS1-201” is a novel long noncoding RNA that has not been studied in the field of AF postablation. Besides there are currently no studies exploring its relationship with immune cells. In addition, the study of the relationship between immune cells and AF, especially the association between NK cells and AF, is still in its early stages. We plan to address these aspects in our future research.

## Conclusion

We discovered that AF recurrence after catheter ablation may be associated with immune responses and fibrosis, with the extracellular matrix playing a crucial role. We identified 9 hub genes (*IGF2*, *FGFR1*, *MMP9*, *HSPG2*, *GZMB*, *PEG10*, *GNLY*, *COL6A1*, and *KCNE3*) and constructed a lncRNA‒mRNA regulatory network. TMEM51-AS1-201, along with its targeted hub genes, may be potential vital targets for AF recurrence postablation.

### Supplementary Information

**Additional file 1: Table S1.** Sequences of primers used for qRT-PCR. **Table S2. **Differentially expressed genes. **Table S3. **Differentially expressed lnc-RNAs.

## Data Availability

All clean data files are publicly available in the GEO database [39] under the accession number GSE245886.

## Abbreviations

AF: Atrial fibrillation
P-RAF: Presence of recurrence after AF ablation
A-RAF: Absence of recurrence after AF ablation
DEGs: Differentially expressed genes
DE-lncRNA: Differentially expressed long noncoding RNA
GO: Gene ontology
KEGG: Kyoto Encyclopedia of Genes and Genomes
PPI: Protein–protein interactions
qRT-PCR: Real-time quantitative PCR
BP: Biological processes
CC: Cellular components
MF: Molecular functions
