# Supplementary material for: Analyses of lncRNA and mRNA profiles in recurrent atrial fibrillation after catheter ablation
Source: Eur J Med Res. 2024 Apr 20;29:244. doi: 10.1186/s40001-024-01799-3 (PMC11031869; doi:10.1186/s40001-024-01799-3)
Supplement: Supplementary file 1 — Additional file 1: Table S1. Sequences of primers used for qRT-PCR. Table S2. Differentially expressed genes. Table S3. Differentially expressed lnc-RNAs. [file 40001_2024_1799_MOESM1_ESM.docx]

**Additional file Tables**

**Additional file Table S1.** Sequences of primers used for qRT-PCR

| Gene | Primer | Sequence(5'to3') |
| --- | --- | --- |
| β-actin | Forward | GGCACCCAGCACAATGAA |
|  | Reverse | GGCACCCAGCACAATGAA |
| FGFR1 | Forward | AGATGGACACCTGCTTCAC |
|  | Reverse | ATGGCAAGGAGGAACAGAG |
| MMP9 | Forward | GTCTTCCAGTACCGAGAG |
|  | Reverse | AGGATGTCATAGGTCACG |
| GNLY | Forward | CTTGTCCTGTGGAAGAAG |
|  | Reverse | AGACTGGAGAGTGGATTC |
| COL6A1 | Forward | CCCACCAATCCTCACCTAA |
|  | Reverse | CGCCGAGATTTATGTCTGC |
| HSPG2 | Forward | CAACAGACAACGGACGGA |
|  | Reverse | GGGCAGGGTGGTCATATC |
| IGF2 | Forward | AACACTGAATGTCACCTGT |
|  | Reverse | GCGTTAAAGGAGTTGAGTTG |
| TMEM51-AS1-201 | Forward | GCTGAGACCGACTGATTA |
|  | Reverse | GCCTCCTTTCCCTGTAAT |

*Abbreviations: qRT-PCR, quantitative real-time polymerase chain reaction.*

**Additional file Table S2.** Differentially expressed genes

| #ID | gene_name | Pvalue | log2FC | regulated |
| --- | --- | --- | --- | --- |
| ENSG00000116729 | WLS | 0.006277025 | 1.303636175 | up |
| ENSG00000075234 | TTC38 | 0.00068862 | 0.802580109 | up |
| ENSG00000157514 | TSC22D3 | 0.006985714 | 0.649070329 | up |
| ENSG00000196428 | TSC22D2 | 0.001128042 | 1.719977936 | up |
| ENSG00000249992 | TMEM158 | 0.001180007 | 2.48958455 | up |
| ENSG00000166548 | TK2 | 0.001635175 | 0.843883951 | up |
| ENSG00000088992 | TESC | 0.003890657 | 0.928382828 | up |
| ENSG00000164674 | SYTL3 | 0.000033558 | 0.728617048 | up |
| ENSG00000259332 | ST20-MTHFS | 0.007099911 | 0.880049931 | up |
| ENSG00000159674 | SPON2 | 0.000366297 | 0.949784029 | up |
| ENSG00000235169 | SMIM1 | 0.007320917 | 1.223406287 | up |
| ENSG00000101082 | SLA2 | 0.000492272 | 0.641747557 | up |
| ENSG00000179526 | SHARPIN | 0.007680256 | 0.798689013 | up |
| ENSG00000027869 | SH2D2A | 0.000467823 | 0.605663201 | up |
| ENSG00000198574 | SH2D1B | 0.00107718 | 0.883604308 | up |
| ENSG00000141622 | RNF165 | 0.000139193 | 1.099182938 | up |
| ENSG00000107317 | PTGDS | 0.002311039 | 1.576132069 | up |
| ENSG00000180644 | PRF1 | 0.000404196 | 0.876707517 | up |
| ENSG00000166289 | PLEKHF1 | 0.00300935 | 0.588037174 | up |
| ENSG00000241360 | PDXP | 0.008211145 | 0.912283068 | up |
| ENSG00000130558 | OLFM1 | 0.001565762 | 1.46739712 | up |
| ENSG00000135114 | OASL | 0.001609062 | 1.006663078 | up |
| ENSG00000154146 | NRGN | 0.008944542 | 1.003794826 | up |
| ENSG00000103148 | NPRL3 | 0.003836581 | 1.070273157 | up |
| ENSG00000110400 | NECTIN1 | 0.004021845 | 0.899002172 | up |
| ENSG00000189430 | NCR1 | 0.000091937 | 0.872508212 | up |
| ENSG00000117400 | MPL | 0.008221726 | 1.004145885 | up |
| ENSG00000100985 | MMP9 | 0.006244261 | 1.499022598 | up |
| ENSG00000100427 | MLC1 | 0.004659065 | 0.844769748 | up |
| ENSG00000176845 | METRNL | 0.002103511 | 0.911287976 | up |
| ENSG00000181350 | LRRC75A | 0.002484525 | 1.234638597 | up |
| ENSG00000221957 | KIR2DS4 | 0.001448978 | 1.240568774 | up |
| ENSG00000125498 | KIR2DL1 | 0.002555525 | 1.668676519 | up |
| ENSG00000180509 | KCNE1 | 0.008552669 | 0.872603943 | up |
| ENSG00000140044 | JDP2 | 0.006813255 | 0.906746654 | up |
| ENSG00000259207 | ITGB3 | 0.009221076 | 0.810284097 | up |
| ENSG00000185950 | IRS2 | 0.007877349 | 1.437067345 | up |
| ENSG00000115602 | IL1RL1 | 0.001961638 | 1.239439177 | up |
| ENSG00000198502 | HLA-DRB5 | 0.001085803 | 1.679175608 | up |
| ENSG00000206177 | HBM | 0.00637165 | 1.514147733 | up |
| ENSG00000100453 | GZMB | 0.000000247 | 1.087884984 | up |
| ENSG00000158292 | GPR153 | 0.003704045 | 1.050029814 | up |
| ENSG00000115523 | GNLY | 0.000209142 | 1.07662838 | up |
| ENSG00000165702 | GFI1B | 0.008495724 | 0.867526613 | up |
| ENSG00000137441 | FGFBP2 | 0.000486664 | 0.829467712 | up |
| ENSG00000173040 | EVC2 | 0.002405192 | 2.163349513 | up |
| ENSG00000141736 | ERBB2 | 0.000463649 | 0.853010116 | up |
| ENSG00000171617 | ENC1 | 0.000316413 | 0.680648932 | up |
| ENSG00000177613 | CSTF2T | 0.000044174 | 1.594199899 | up |
| ENSG00000103196 | CRISPLD2 | 0.005378435 | 0.932712789 | up |
| ENSG00000120885 | CLU | 0.009181564 | 0.742884786 | up |
| ENSG00000175040 | CHST2 | 0.006384382 | 0.592256523 | up |
| ENSG00000166664 | CHRFAM7A | 0.000854472 | 0.947833844 | up |
| ENSG00000172216 | CEBPB | 0.004411385 | 1.558238445 | up |
| ENSG00000007080 | CCDC124 | 0.008828665 | 0.781974964 | up |
| ENSG00000173068 | BNC2 | 0.007106573 | 1.006913799 | up |
| ENSG00000158470 | B4GALT5 | 0.005006092 | 0.721377636 | up |
| ENSG00000163635 | ATXN7 | 0.008998049 | 0.767184547 | up |
| ENSG00000054793 | ATP9A | 0.005155019 | 1.037118138 | up |
| ENSG00000205336 | ADGRG1 | 0.005073606 | 0.737132547 | up |
| ENSG00000284874 | AC000093.1 | 0.00019479 | 1.718540906 | up |
| ENSG00000165029 | ABCA1 | 0.004890667 | 0.838936597 | up |
| ENSG00000197134 | ZNF257 | 0.003486056 | -1.028951735 | down |
| ENSG00000176293 | ZNF135 | 0.006643208 | -1.362663584 | down |
| ENSG00000104427 | ZC2HC1A | 0.00651286 | -0.995338323 | down |
| ENSG00000137831 | UACA | 0.009200027 | -1.402008741 | down |
| ENSG00000198467 | TPM2 | 0.001165173 | -4.279467887 | down |
| ENSG00000130598 | TNNI2 | 0.001850364 | -3.362431928 | down |
| ENSG00000265681 | RPL17 | 0.007212148 | -0.825147546 | down |
| ENSG00000151490 | PTPRO | 0.007576717 | -0.605721499 | down |
| ENSG00000134247 | PTGFRN | 0.00011418 | -1.430066049 | down |
| ENSG00000242265 | PEG10 | 0.004521117 | -2.987008138 | down |
| ENSG00000105185 | PDCD5 | 0.000191629 | -0.682664224 | down |
| ENSG00000169918 | OTUD7A | 0.000195676 | -0.725589584 | down |
| ENSG00000140876 | NUDT7 | 0.00739481 | -0.804449164 | down |
| ENSG00000164978 | NUDT2 | 0.005215521 | -0.659867075 | down |
| ENSG00000169992 | NLGN2 | 0.009308897 | -1.114377397 | down |
| ENSG00000184613 | NELL2 | 0.00000439 | -0.860514694 | down |
| ENSG00000109063 | MYH3 | 0.000706763 | -4.317161388 | down |
| ENSG00000104177 | MYEF2 | 0.004319908 | -1.32688921 | down |
| ENSG00000106484 | MEST | 0.001386384 | -2.570874865 | down |
| ENSG00000173114 | LRRN3 | 0.007592502 | -0.782420127 | down |
| ENSG00000150477 | KIAA1328 | 0.000198427 | -0.654838345 | down |
| ENSG00000175538 | KCNE3 | 0.00706557 | -1.393657269 | down |
| ENSG00000109944 | JHY | 0.001398009 | -0.895858715 | down |
| ENSG00000167244 | IGF2 | 0.00172098 | -5.519238848 | down |
| ENSG00000142798 | HSPG2 | 0.009744169 | -1.747693718 | down |
| ENSG00000073605 | GSDMB | 0.003860798 | -0.613144728 | down |
| ENSG00000077782 | FGFR1 | 0.004894623 | -1.192291369 | down |
| ENSG00000026103 | FAS | 0.00127506 | -0.60076815 | down |
| ENSG00000151491 | EPS8 | 0.001458805 | -1.417447484 | down |
| ENSG00000203965 | EFCAB7 | 0.009651092 | -0.893290394 | down |
| ENSG00000114942 | EEF1B2 | 0.004695226 | -0.638066107 | down |
| ENSG00000271079 | CTAGE15 | 0.000264834 | -1.741530298 | down |
| ENSG00000142156 | COL6A1 | 0.004605996 | -2.996559364 | down |
| ENSG00000186265 | BTLA | 0.008397595 | -0.850597818 | down |

**Additional file Table S3.** Differentially expressed lnc-RNAs.

| #ID | P-value | log2FC | regulated |
| --- | --- | --- | --- |
| ENST00000366153 | 0.002187 | -1.66773 | down |
| ENST00000435624 | 0.004318 | -1.452 | down |
| ENST00000446423 | 0.000119 | -1.60677 | down |
| ENST00000447009 | 0.007864 | -2.36369 | down |
| ENST00000482677 | 0.00613 | -2.12709 | down |
| ENST00000499131 | 0.00874 | -2.1096 | down |
| ENST00000529656 | 0.007476 | -0.93481 | down |
| ENST00000544657 | 9.27E-06 | -4.54809 | down |
| ENST00000544890 | 0.009698 | -1.9889 | down |
| ENST00000553415 | 0.000716 | -1.1687 | down |
| ENST00000564771 | 0.001759 | -0.94604 | down |
| ENST00000565297 | 0.007122 | -1.40615 | down |
| ENST00000568885 | 0.001069 | -1.13828 | down |
| ENST00000575890 | 0.008746 | -0.91432 | down |
| ENST00000578802 | 0.006649 | -2.02311 | down |
| ENST00000581913 | 0.000787 | -4.84316 | down |
| ENST00000587759 | 2.14E-05 | -4.21254 | down |
| ENST00000602843 | 0.002858 | -1.23581 | down |
| ENST00000605534 | 0.006437 | -1.84473 | down |
| ENST00000607781 | 0.005342 | -1.55802 | down |
| ENST00000608783 | 0.002 | -1.586 | down |
| ENST00000611728 | 0.007289 | -1.19141 | down |
| ENST00000619110 | 0.007053 | -0.66839 | down |
| ENST00000621019 | 0.002388 | -1.30483 | down |
| ENST00000623684 | 0.002897 | -2.13575 | down |
| MSTRG.101198.2 | 0.000679 | -2.6071 | down |
| MSTRG.101402.1 | 0.002537 | -3.00533 | down |
| MSTRG.103513.1 | 0.005588 | -1.14397 | down |
| MSTRG.103939.1 | 0.002906 | -1.62955 | down |
| MSTRG.104834.31 | 0.000556 | -1.705 | down |
| MSTRG.105196.1 | 0.009361 | -2.37954 | down |
| MSTRG.10542.3 | 1.02E-10 | -7.01064 | down |
| MSTRG.106510.4 | 0.0014 | -1.72095 | down |
| MSTRG.106627.5 | 0.003225 | -0.68951 | down |
| MSTRG.109138.20 | 0.008694 | -0.782 | down |
| MSTRG.109139.1 | 0.004004 | -0.9332 | down |
| MSTRG.109721.1 | 0.009177 | -2.63901 | down |
| MSTRG.10992.8 | 0.002477 | -4.54348 | down |
| MSTRG.111516.1 | 7.92E-05 | -0.93992 | down |
| MSTRG.21044.9 | 0.001016 | -1.64966 | down |
| MSTRG.21338.1 | 0.004876 | -0.84197 | down |
| MSTRG.23391.2 | 0.006154 | -0.6462 | down |
| MSTRG.23762.1 | 0.007892 | -1.00566 | down |
| MSTRG.26033.47 | 2.80E-06 | -2.37084 | down |
| MSTRG.28382.17 | 3.94E-10 | -6.77484 | down |
| MSTRG.28854.1 | 0.001381 | -1.15983 | down |
| MSTRG.29491.1 | 0.00583 | -1.86809 | down |
| MSTRG.29731.2 | 0.000237 | -3.90246 | down |
| MSTRG.30823.1 | 0.000497 | -2.6564 | down |
| MSTRG.31531.1 | 0.002799 | -1.95239 | down |
| MSTRG.38802.1 | 0.006124 | -2.38439 | down |
| MSTRG.39372.1 | 0.009429 | -2.9305 | down |
| MSTRG.39950.1 | 0.000228 | -3.23477 | down |
| MSTRG.41451.1 | 0.000597 | -0.97239 | down |
| MSTRG.43684.1 | 0.001075 | -0.69708 | down |
| MSTRG.47516.1 | 0.005942 | -1.23396 | down |
| MSTRG.48273.1 | 0.003273 | -2.94346 | down |
| MSTRG.49976.1 | 0.004673 | -1.06088 | down |
| MSTRG.50441.4 | 0.001072 | -1.30157 | down |
| MSTRG.51161.8 | 0.008734 | -1.1576 | down |
| MSTRG.52125.1 | 0.000137 | -1.96306 | down |
| MSTRG.5698.1 | 0.003799 | -1.19552 | down |
| MSTRG.5739.50 | 0.000363 | -1.83566 | down |
| MSTRG.57588.1 | 0.000341 | -3.25344 | down |
| MSTRG.57763.13 | 0.003767 | -0.95109 | down |
| MSTRG.5781.1 | 0.005066 | -2.1568 | down |
| MSTRG.58945.1 | 0.000702 | -1.8576 | down |
| MSTRG.59222.1 | 0.005363 | -2.7421 | down |
| MSTRG.59334.4 | 0.004942 | -0.78237 | down |
| MSTRG.65281.1 | 0.000365 | -2.27859 | down |
| MSTRG.66539.12 | 1.48E-05 | -5.28808 | down |
| MSTRG.66824.2 | 9.93E-06 | -1.86702 | down |
| MSTRG.67512.1 | 0.004713 | -1.01285 | down |
| MSTRG.67997.2 | 0.005723 | -1.53855 | down |
| MSTRG.69259.1 | 5.88E-05 | -1.09823 | down |
| MSTRG.72272.1 | 0.005546 | -1.69077 | down |
| MSTRG.7434.12 | 0.000362 | -3.71608 | down |
| MSTRG.7762.1 | 0.004886 | -1.72609 | down |
| MSTRG.79028.2 | 0.004648 | -2.06052 | down |
| MSTRG.79418.8 | 0.002546 | -0.89322 | down |
| MSTRG.85206.1 | 0.003463 | -1.43166 | down |
| MSTRG.8565.1 | 0.006676 | -1.23127 | down |
| MSTRG.86673.1 | 0.002269 | -0.8464 | down |
| MSTRG.86742.6 | 0.001886 | -1.45646 | down |
| MSTRG.87349.1 | 0.004295 | -0.80604 | down |
| MSTRG.88597.3 | 0.002031 | -2.66987 | down |
| MSTRG.89428.64 | 0.005357 | -1.66586 | down |
| MSTRG.90441.2 | 0.000212 | -2.86651 | down |
| MSTRG.93130.36 | 0.002276 | -1.31566 | down |
| MSTRG.94694.2 | 0.00894 | -1.23641 | down |
| MSTRG.94830.36 | 0.009093 | -0.79222 | down |
| MSTRG.95718.15 | 0.006141 | -1.82794 | down |
| MSTRG.97801.1 | 0.000848 | -3.40988 | down |
| MSTRG.98340.1 | 0.009213 | -2.56206 | down |
| MSTRG.99842.25 | 0.003722 | -1.8584 | down |
| ENST00000332012 | 0.002368 | 4.184859 | up |
| ENST00000418006 | 0.000197 | 3.610418 | up |
| ENST00000424235 | 0.006074 | 2.0877 | up |
| ENST00000430166 | 0.002814 | 3.333488 | up |
| ENST00000433669 | 0.000512 | 2.695656 | up |
| ENST00000440406 | 2.03E-06 | 2.743844 | up |
| ENST00000451646 | 0.00607 | 1.528351 | up |
| ENST00000451762 | 0.009879 | 1.214303 | up |
| ENST00000461448 | 1.99E-06 | 2.383366 | up |
| ENST00000500148 | 0.000486 | 3.327023 | up |
| ENST00000507444 | 0.002212 | 2.271758 | up |
| ENST00000520515 | 0.00622 | 0.708607 | up |
| ENST00000524264 | 0.004051 | 1.4988 | up |
| ENST00000554614 | 0.006138 | 0.941486 | up |
| ENST00000562232 | 0.009866 | 4.605361 | up |
| ENST00000565797 | 0.003774 | 0.704453 | up |
| ENST00000565978 | 0.004774 | 1.993559 | up |
| ENST00000568395 | 0.001642 | 5.163096 | up |
| ENST00000570515 | 0.000234 | 10.25738 | up |
| ENST00000574387 | 0.00124 | 4.060621 | up |
| ENST00000576810 | 7.25E-09 | 11.93691 | up |
| ENST00000582267 | 0.002046 | 1.981866 | up |
| ENST00000591299 | 0.002784 | 2.540143 | up |
| ENST00000592381 | 0.00803 | 1.452451 | up |
| ENST00000607453 | 0.001878 | 3.062987 | up |
| ENST00000608056 | 0.003234 | 2.31676 | up |
| ENST00000608605 | 8.12E-06 | 2.680601 | up |
| ENST00000609598 | 0.002085 | 2.654771 | up |
| ENST00000609680 | 0.006881 | 1.983822 | up |
| ENST00000618309 | 8.19E-05 | 2.121092 | up |
| ENST00000623095 | 0.008611 | 2.287017 | up |
| ENST00000647388 | 0.007527 | 3.321178 | up |
| ENST00000648075 | 0.007633 | 2.991359 | up |
| ENST00000648264 | 0.000114 | 2.154353 | up |
| MSTRG.100616.3 | 0.00278 | 2.368691 | up |
| MSTRG.102077.20 | 0.00291 | 1.997561 | up |
| MSTRG.105396.1 | 0.007686 | 2.996705 | up |
| MSTRG.107871.24 | 0.009102 | 1.0033 | up |
| MSTRG.109254.2 | 0.000932 | 1.356222 | up |
| MSTRG.10972.1 | 0.004835 | 2.289337 | up |
| MSTRG.1098.59 | 0.007516 | 0.956995 | up |
| MSTRG.111196.5 | 0.004991 | 1.367044 | up |
| MSTRG.11255.1 | 0.000138 | 1.255091 | up |
| MSTRG.11514.1 | 0.001083 | 2.506036 | up |
| MSTRG.12610.1 | 0.003958 | 1.296742 | up |
| MSTRG.13045.6 | 0.001693 | 1.077377 | up |
| MSTRG.13505.1 | 8.59E-05 | 3.325183 | up |
| MSTRG.14485.40 | 0.003661 | 4.070032 | up |
| MSTRG.1511.1 | 0.000218 | 4.575579 | up |
| MSTRG.15192.1 | 0.005311 | 1.359648 | up |
| MSTRG.16556.25 | 7.29E-16 | 3.837415 | up |
| MSTRG.18074.1 | 0.002686 | 1.138105 | up |
| MSTRG.20574.1 | 0.0027 | 1.135099 | up |
| MSTRG.23492.30 | 0.001956 | 2.429395 | up |
| MSTRG.24843.1 | 0.006805 | 1.998516 | up |
| MSTRG.26406.1 | 0.004807 | 0.945518 | up |
| MSTRG.28334.12 | 0.005788 | 2.929002 | up |
| MSTRG.32562.1 | 0.000465 | 3.630266 | up |
| MSTRG.33111.5 | 0.000224 | 2.544362 | up |
| MSTRG.33645.1 | 0.009127 | 2.274197 | up |
| MSTRG.3404.1 | 5.91E-05 | 1.250622 | up |
| MSTRG.37191.1 | 0.008162 | 2.159025 | up |
| MSTRG.38808.20 | 0.000821 | 1.643563 | up |
| MSTRG.38889.14 | 0.000716 | 1.979078 | up |
| MSTRG.40683.16 | 0.001576 | 3.151324 | up |
| MSTRG.42614.12 | 0.001565 | 1.119328 | up |
| MSTRG.43594.1 | 0.003387 | 1.139012 | up |
| MSTRG.44707.39 | 0.001038 | 0.801507 | up |
| MSTRG.45662.44 | 0.002583 | 0.934346 | up |
| MSTRG.48778.1 | 0.006598 | 1.56112 | up |
| MSTRG.52994.26 | 0.001318 | 1.465361 | up |
| MSTRG.53170.2 | 0.000958 | 1.282824 | up |
| MSTRG.53238.28 | 0.000166 | 2.309367 | up |
| MSTRG.54177.20 | 0.005912 | 3.197485 | up |
| MSTRG.56497.9 | 0.005192 | 2.017318 | up |
| MSTRG.58283.2 | 0.000523 | 1.530732 | up |
| MSTRG.59525.39 | 0.009997 | 2.592246 | up |
| MSTRG.60296.1 | 4.55E-05 | 0.961777 | up |
| MSTRG.61023.37 | 0.009585 | 3.13099 | up |
| MSTRG.64147.41 | 0.002296 | 5.40233 | up |
| MSTRG.64201.1 | 0.005315 | 1.626157 | up |
| MSTRG.64202.2 | 0.003243 | 1.939022 | up |
| MSTRG.65496.2 | 0.003767 | 3.221802 | up |
| MSTRG.6571.1 | 0.008835 | 0.658323 | up |
| MSTRG.68499.17 | 0.00011 | 8.412949 | up |
| MSTRG.68499.21 | 5.84E-05 | 8.095039 | up |
| MSTRG.69007.5 | 0.003268 | 3.277199 | up |
| MSTRG.72958.2 | 0.00628 | 2.194534 | up |
| MSTRG.73191.1 | 0.009194 | 1.726283 | up |
| MSTRG.73481.1 | 0.002314 | 2.918002 | up |
| MSTRG.7462.34 | 0.006479 | 3.169973 | up |
| MSTRG.7514.35 | 0.003557 | 1.006297 | up |
| MSTRG.76817.1 | 0.009917 | 2.15573 | up |
| MSTRG.77296.1 | 0.006119 | 1.041343 | up |
| MSTRG.78006.1 | 0.001719 | 1.530889 | up |
| MSTRG.81144.2 | 0.001098 | 2.7385 | up |
| MSTRG.82919.1 | 0.00176 | 1.850718 | up |
| MSTRG.82935.1 | 0.009636 | 1.443537 | up |
| MSTRG.84905.23 | 0.005948 | 1.749394 | up |
| MSTRG.85507.1 | 0.000247 | 1.415615 | up |
| MSTRG.85601.2 | 0.006311 | 1.721481 | up |
| MSTRG.89428.57 | 4.74E-17 | 10.35613 | up |
| MSTRG.90668.1 | 0.002811 | 1.054066 | up |
| MSTRG.91357.1 | 0.006767 | 1.151147 | up |
| MSTRG.95567.1 | 0.005177 | 1.041744 | up |
| MSTRG.96360.14 | 0.005593 | 1.823685 | up |
| MSTRG.96363.1 | 0.003929 | 1.785194 | up |
| MSTRG.97768.2 | 0.001398 | 1.913816 | up |
